# Supplementary figures and images for: A Potential Biomarker of Brain Activity in Autism Spectrum Disorders: A Pilot fNIRS Study in Female Preschoolers
Source: Brain Sci. 2023 Jun 14;13(6):951. doi: 10.3390/brainsci13060951 (PMC10296408; doi:10.3390/brainsci13060951)

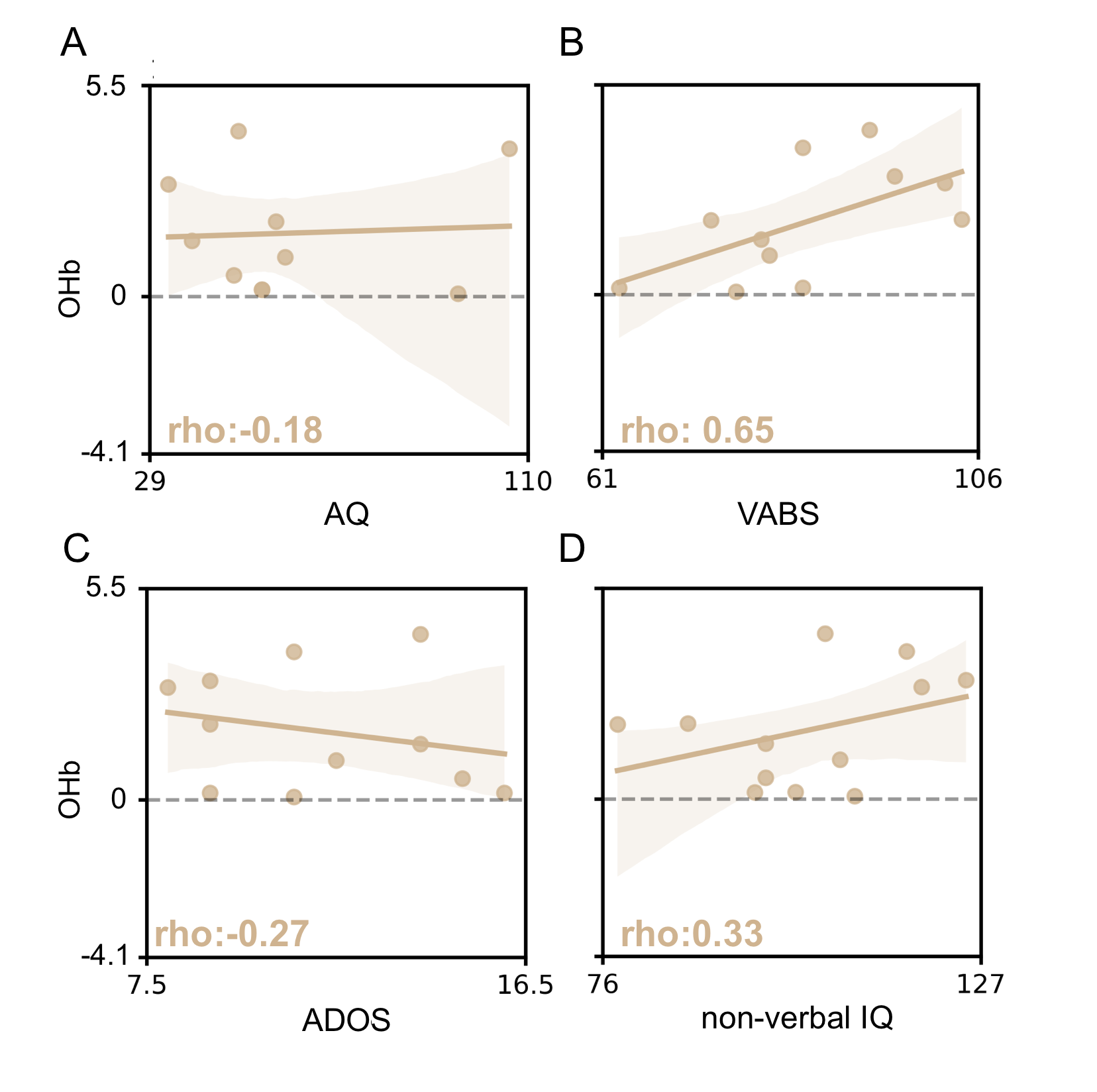

Supplement: Supplementary file 1 [file brainsci-13-00951-s001.zip › Figure S1.tiff]

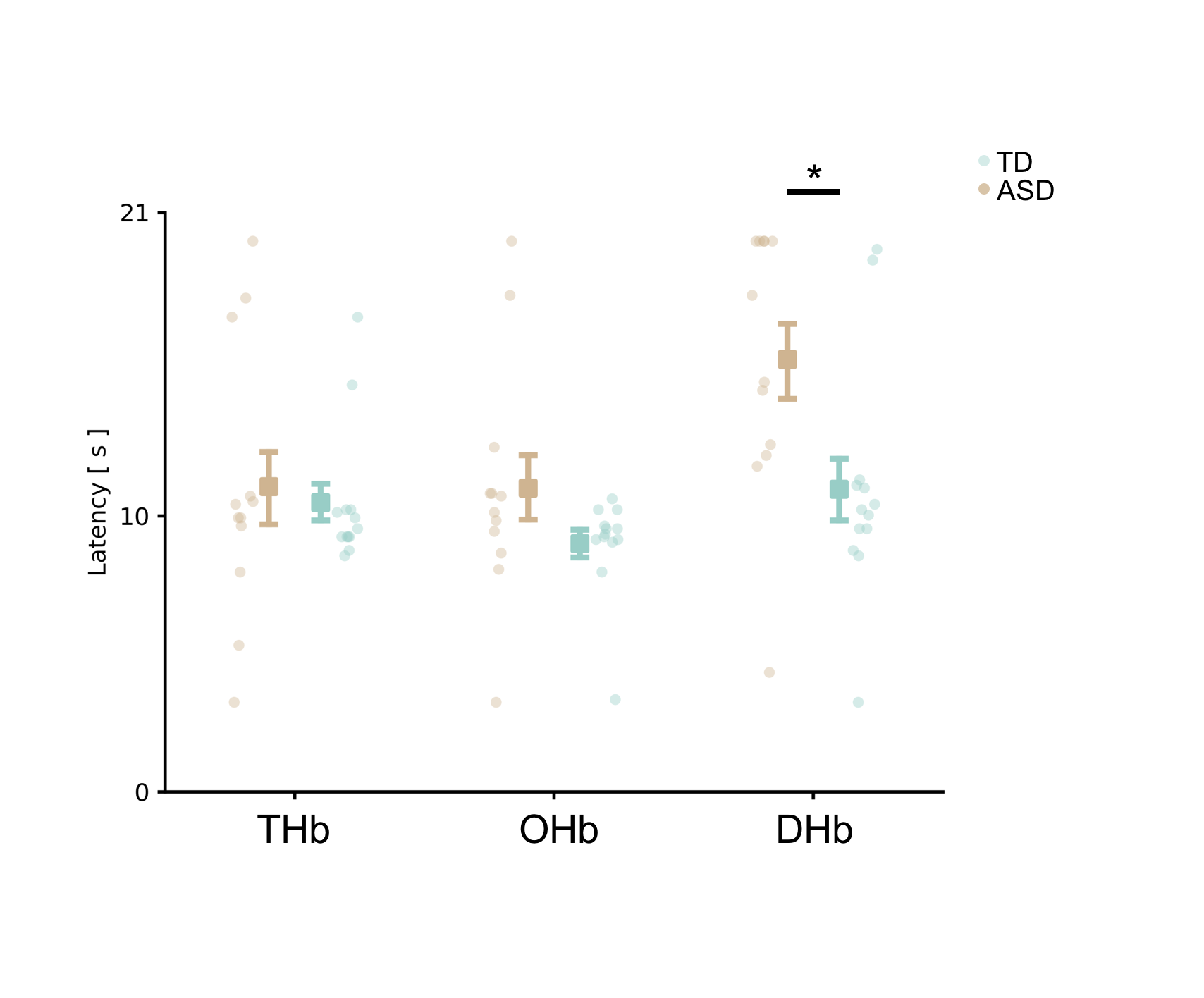

Supplement: Supplementary file 1 [file brainsci-13-00951-s001.zip › Figure S2.tiff]
